# Exploring Students Online Learning Behavioral Engagement in University: Factors, Academic Performance and Their Relationship

**DOI:** 10.3390/bs15010078

**Published:** 2025-01-17

**Authors:** Yonghong Wang, Mingzhang Zuo, Xiangchun He, Zhifeng Wang

**Affiliations:** 1 Faculty of Artificial Intelligence in Education, Central China Normal University, Wuhan 430079, China; wangyh4437@nwnu.edu.cn (Y.W.); mzzuo@mail.ccnu.edu.cn (M.Z.); 2 College of Educational Technology, Northwest Normal University, Lanzhou 730070, China; hxc@nwnu.edu.cn

**Keywords:** online learning behavioral engagement, evaluation indicators, Delphi method, analytic hierarchy process, academic performance

## Abstract

As online learning platforms become prevalent, online learning has been an important way for college students. Online learning engagement, as an evaluation of online learning quality, is crucial for enhancing learning quality and promoting higher education by investigating college students’ engagement and its influencing factors in the online learning environment. This paper aims to identify key factors affecting college students’ online learning behavioral engagement. Based on a literature review, the Delphi expert consultation method was used to build an assessment framework covering five dimensions (participation, concentration, interaction, challenge, and self-monitoring) with sixteen specific indicators. The Analytic Hierarchy Process (AHP) determined the weights of these factors. Then, data from 63 students using the “Ketangpai” online learning platform were collected and analyzed to explore the correlation and predictive relationships between online learning behavior indicators and academic achievements. The results showed a strong correlation between the frequency of accessing online learning resources and long-term online learning and academic performance, and a prediction model was established. The framework offers theoretical and methodological insights for designing online learning activities and evaluating learning quality. It supports intervening in and assessing college students’ online learning processes and improving learning quality. Also, exploring the relationship helps educators formulate personalized online teaching strategies, improving online education effectiveness and students’ learning experiences.

## 1. Introduction

With the in-depth application of information technology in educational scenarios, students’ traditional learning methods have been significantly influenced. Online learning has become a common learning mode for college students. This has led to the emergence of online learning platforms such as MOOCs, Coursera, and edX while virtual learning communities are transforming traditional teaching models and methods. Globally, higher education institutions have witnessed a paradigm shift towards online instruction, emphasizing the importance of online learning, especially for students who are unable to attend in-person classes (Roque-Hernández et al., 2021). Learning engagement is a key factor influencing the quality of learners’ online learning (Kop et al., 2011). Consequently, enhancing learners’ engagement in large-scale online education has become a central issue for educators and researchers. Nevertheless, quality issues such as “low engagement” and “low cognitive level of learning” remain prevalent in online courses. Both domestic and international educational sectors have placed great emphasis on the quality of online learning, which is largely contingent upon learners’ engagement (Coates, 2010). Due to the unique characteristics of online learning, learners in online learning environments need to exert more cognitive effort to handle complex learning situations compared to those in offline learning environments (Akyol & Garrison, 2011). However, quality crises such as “low engagement” and “shallow cognitive level of learning” still pervade online courses (Yin & Xu, 2016). At present, both domestic and international communities attach great importance to the quality of online learning, which depends to a large extent on learners’ learning engagement (Coates, 2010). Behavioral engagement is a fundamental dimension of learning engagement and acts as a conduit for students’ psychological engagement (affective and cognitive engagement) in learning (Newmann, 1992). Learning behavioral engagement, a critical factor influencing academic performance, demands timely assessment and intervention to enhance overall academic outcomes (Johnson & Sinatra, 2013). Evidently, online learning behavioral engagement is a significant factor affecting online academic performance. Investigating the components of online learning behavioral engagement and its relationship with academic performance can effectively promote the improvement of students’ online academic performance. The evolving technological landscape and dynamic learning environments have a profound impact on student engagement, necessitating a deeper exploration of its constructs in the online context to enhance student learning outcomes (Al Mamun & Lawrie, 2023).

Relevant researchers have explored online learning engagement from diverse perspectives. Nandi et al. (2012) discovered that students’ inclination to participate in discussions within online learning environments is highly influenced by teacher behavior. Hew et al. (2010) argued that online learning behaviors encompass learners’ discussion, identification, and response situations. They have found that online learning engagement is equally predictive of academic performance (Macfadyen & Dawson, 2010; Wong, 2013; Hamane, 2014). Online learning engagement, as an essential part of a student’s learning process and a presentation of students’ learning self-efficacy, plays a positive role in students’ academic achievement (Luan et al., 2021). With the increasing prominence of online learning in higher education, the connection between online learning engagement and students’ academic performance has drawn more attention (Rajabalee & Santally, 2021). Many studies have explored student engagement in online learning at tertiary institutions and verified its impact on academic performance (Johar et al., 2023). Despite the widespread recognition of the influence of online learning engagement on academic performance, there is a scarcity of studies exploring the underlying mechanisms of this relationship. Moreover, the analysis and evaluation of online learning behavioral engagement do not sufficiently refer to the results of learning engagement research regarding the composition, types, and assessment of behavioral engagement. A more comprehensive framework for online learning behavioral assessment has not been formed, and there is a lack of empirical research on the assessment indicators of behavioral engagement. Especially for the college student group, it is of great importance to explore the key behavioral factors that influence their online learning and analyze the relationship between online learning behavioral indicators and academic achievements through empirical research. Therefore, more empirical research is required to investigate how students’ engagement in learning management systems can be analyzed, which behavioral indicators can effectively characterize engagement, and how online learning behavioral engagement affects academic performance.

Student engagement and academic achievement play crucial roles in an online learning environment (Berman & Artino, 2018). Nevertheless, the insufficient communication between teachers and students results in less-than-satisfactory student participation in online learning, where students exhibit deficiencies in terms of persistence and efficiency. This study aimed to overcome the limitations of existing research and conduct a comprehensive exploration of the effectiveness of online learning. Specifically, the study intended to explore the key factors influencing college students’ online learning behavioral engagement and the influence mechanism between each behavioral indicator and academic achievement. The Delphi method involves collecting expert opinions through a back-and-forth communication process. After several rounds of consultation, the experts’ predictions tend to converge (Green et al., 1999). To develop and revise the evaluation framework for college students’ online learning behavioral engagement, expert consultation was used to enhance the scientific validity of the evaluation indicators and assign weights to them. This study employed the Delphi method for expert consultation, collecting expert opinions to revise and form a comprehensive evaluation framework for college students’ online learning behavioral engagement.

## 2. Literature Review

### 2.1. Online Learning Engagement

Online learning refers to a learning environment that uses the Internet and other technological devices and tools for instructional delivery and the management of academic programs (Barrot et al., 2021). Although it has various benefits in terms of convenience and flexibility (Isaac et al., 2019; Zapata-Cuervo et al., 2022), student online academic performance is not consistently satisfactory. In early research, Tyler (1989) proposed, from the perspective of work engagement, that the greater the degree of people’s engagement in work was, the more substantial the rewards would be. A significant assessment for teaching students in a digital environment is engagement (Kobicheva, 2022). Wang et al. (2020) define engagement as a student’s active involvement and interactions with learning activities, processes, and contexts and conceptualize it as a multidimensional construct consisting of behavioral, cognitive, and affective aspects. Bond and Bedenlier (2019) proposed a theoretical framework for engagement in online learning, which includes cognitive engagement, affective engagement, and behavioral engagement, the types of interactions that may influence these dimensions; each dimension of engagement comprises a range of indicators and the potential short-term and long-term outcomes. Based on the factors most directly related to the learning process, Martin (2008) focused learning engagement on two dimensions, namely cognitive engagement and behavioral engagement. Learning engagement, which includes positive, fulfilling, and learning-related states of mind during the learning process, is a multifaceted concept consisting of behavioral, social, cognitive, and conceptual affective dimensions (Jung & Lee, 2018; Sinha et al., 2015). This concept has been acknowledged as a critical indicator in evaluating online courses due to its connection with the quality of online education and students’ performance (Wang et al., 2022). Scholars frequently define learning engagement from a psychological perspective, specifying three dimensions: cognitive, behavioral, and emotional. For example, Newmann (1992) defines learning engagement as “the psychological engagement and effort that students invest in learning, understanding, and mastering knowledge, skills, and technologies to be facilitated in their academic studies”. This comprehensive definition includes not only behavioral engagement but also psychological engagement.

Due to the rapid development of online learning, the engagement in online learning has attracted increasing attention from researchers at home and abroad. Martin and Borup (2022) synthesized, from the literature, five online environmental factors that promote learner engagement, including communication, interaction, presence, collaboration, and community. This emphasizes students’ emotional and behavioral investment in initiating and carrying out learning activities as an essential part of achieving positive learning outcomes. Student engagement is a multifaceted construct that reflects students’ efforts in learning activities and is influenced by various factors within the learning environment. The challenge of maintaining academic success, achievement, and engagement at higher education institutions (HEIs) remains global. Therefore, studies that investigate the relationship between students’ engagement and academic performance in online learning settings should be emphasized (Muir et al., 2019). In higher education, the engagement of online students has implications for important outcomes, including student satisfaction, the perception of learning, and learning persistence (Bolliger & Halupa, 2018; Jung & Lee, 2018). Despite its importance, the literature on student engagement in online learning is relatively scarce (Paulsen & McCormick, 2020). Most scholars concur that behavioral engagement acts as the vehicle for cognitive and emotional engagement, with students showing engagement in both the behavioral and cognitive aspects of the learning process. After integrating the definitions of learning engagement from various scholars, it is generally accepted that it includes three dimensions: behavioral, cognitive, and emotional engagement.

### 2.2. Online Learning Behavioral Engagement

Behavioral engagement pertains to the degree to which students actively participate in online learning activities, such as by listening and reading attentively and engaging in online class discussions (Chiu, 2022; Ding et al., 2017; El-Sayad et al., 2021). Numerous previous studies have indicated that behavioral engagement has a positive impact on academic performance (Bråten et al., 2018; Vermeulen & Volman, 2024). For example, Wang (2017) explored how online behavior engagement affects student achievement in a flipped classroom and discovered that students’ engagement in problem solving activities has a positive influence on their achievement levels. The definition of behavioral engagement is extensive, including the physical behaviors and energy demonstrated by students during learning activities (Borup et al., 2020). For instance, students who exhibit punctuality, actively participate in lectures, respond promptly to teachers’ questions, submit assignments in a timely manner, and show perseverance in the face of difficulties are regarded as behaviorally engaged. Behavioral engagement was stimulated through activities that promote attention and focus, inspire effort, break barriers, and provide flexibility (Vermeulen & Volman, 2024).

In the online learning environment, since learning activities and communications are mediated through technology, a certain degree of technological proficiency is required for behavioral engagement. Martin and Borup (2022) offered a more detailed definition, viewing behavioral engagement as the physical behaviors and energy demonstrated by students during learning activities. This definition not only emphasizes individual behavior and energy but also considers the impact of technology in shaping different forms of behavioral engagement within the online learning context. In online learning, many students believe that learning from online resources on time, participating in peer discussions, increasing the number of posts and replies, and taking quizzes and submitting assignments on time mean that they have been actively engaged in online learning (Liang, 2018). Teachers’ support can indeed improve students’ behavioral engagement in academic activities (Feng et al., 2023).

A large number of studies on online student engagement have mainly concentrated on the behavioral dimension. Engagement has been measured by means of metrics including the quantity of lecture videos watched, participation in discussion forums, and the completion of quizzes and assignments (Hew, 2016; Hui et al., 2019). As behavioral engagement has a greater effect on academic outcomes for students at lower levels of education, additional efforts should be made to increase their motivation for live online learning (Kobicheva, 2022). In this study, the degree of students’ engagement in the learning process was defined by taking into account aspects of behavior, cognition, and emotion. The research specifically focused on behavioral engagement in order to explore the component indicators of college students’ online learning behavioral engagement and its relationship with academic performance.

## 3. Research Question

Through the literature review analysis, behavioral engagement was identified as a key factor influencing students’ online learning engagement, and it serves as a prerequisite for both cognitive and emotional engagement. To identify the key factors that influenced college students’ online learning behavioral engagement and to clarify the complex relationship between online learning behavioral indicators and academic achievement, this study first reviewed the literature to identify the key factors affecting college students’ online learning engagement. It then refined and developed a comprehensive evaluation framework for online learning behavioral engagement through Delphi expert consultation. Next, the study quantitatively analyzed the correlation between online learning behavioral indicators and academic achievement to explore the behavioral indicators that affected college students’ online learning engagement. Finally, a regression model was developed through data analysis to examine the linear relationship and impact degree between online learning behavioral engagement indicators and academic achievement. To guide our investigation, we posed the following two research questions (RQs):

RQ1: What are the online learning behavioral indicators that affect college students’ engagement in online learning?

RQ2: What are the relationships between college students’ online learning behavior indicators and their academic performance?

## 4. Methodology

To achieve the research objectives, the research process primarily involved the development of a college students’ online learning behavioral engagement evaluation framework and the investigation and analysis of the relationships between online learning behavior indicators and academic performance. Specifically, in terms of the evaluation framework development, preliminary evaluation indicators were proposed through a literature review, and these indicators were revised and weighted through expert consultation to form a complete evaluation framework for college students’ online learning behavioral engagement. In the exploration of the relationship, online learning behavior data from college students were collected to analyze their overall online learning behavior characteristics, and the correlations between and predictive power of online learning behavior indicators and academic performance were examined. Ultimately, effective behavioral indicators for predicting college students’ online academic performance were identified, and a regression analysis model was developed.

### 4.1. Developing an Online Learning Behavior Engagement Evaluation Framework for College Students

**Figure 1:**
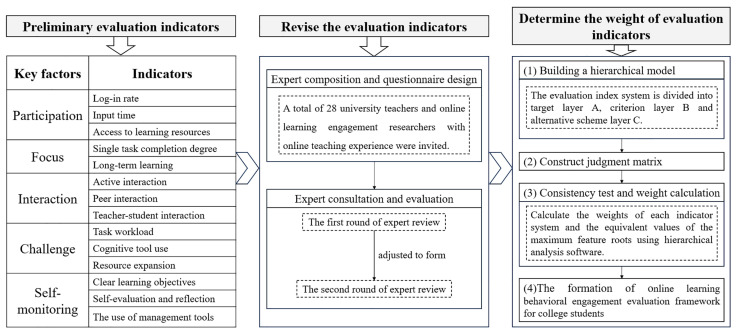
Development process of online learning behavioral engagement evaluation framework for college students.

The development of a comprehensive evaluation framework for online learning behavioral engagement of college students encompassed three crucial stages: the establishment of preliminary evaluation indicators, the revision of evaluation indicators, and the determination of the weight of evaluation indicators. The specific tasks within each stage are depicted in Figure 1.

#### 4.1.1. Preliminary Evaluation Indicators

Behavioral engagement is defined as effort and participation, or students’ involvement in learning activities (Fredricks et al., 2004). Atherton et al. (2017) indicated that the more frequently students access and interact with learning materials, the better their academic performance will be. Therefore, students’ engagement in online learning merits further investigation to determine its impact on their learning. Participation pertains to the time and energy that students expend on the fundamental activities of a course, namely the behaviors by which students comply with and react to course regulations and teachers’ requirements. Some studies have further defined participation in the prescribed tasks related to course learning evaluation as performance engagement, including timely submission of assignments, completion of assignments, and punctual attendance at exams (Appleton et al., 2008; Lam et al., 2014). The behavioral engagement subscale developed by Lam et al. (2014) was utilized to explore the dimensions of active participation, concentration, and persistence. Participation-related engagement is the fundamental behavioral engagement in course learning, reflecting the degree of students’ acceptance and approval of course rules and requirements and serving as the basis for other behavioral engagement. Some scholars have suggested that behavioral engagement is defined as the observable behaviors, including effort, concentration, persistence, attention, and hard work, that students display during their participation in learning (Redmond et al., 2018). Therefore, “participation” and “focus” were identified as two key behaviors influencing online learning engagement and were established as evaluation indicators.

Engagement does not exist in a vacuum but instead emerges out of the interaction between the learners, the learning contexts, and learning outcomes (Skinner, 2016; Wang et al., 2020). Furthermore, Moore’s framework identifies the essential types of interactions necessary for effective learning, highlighting their significance in educational settings (Abou-Khalil et al., 2021). Incorporating various learning theories and promoting student engagement through effective interaction and technology-based pedagogy can enhance the online learning experience and contribute to successful learning outcomes (Tarazi & Ruiz-Cecilia, 2023). Previous studies found that students who interact frequently and meaningfully with course materials are more likely to achieve success (Abou-Khalil et al., 2021; Malan, 2020). Apparently, persistence mainly represents the continuous efforts exerted by students in pursuit of learning goals in the face of difficulties, problems, and pressure. Meanwhile, concentration refers to the extent to which students focus their attention on the learning content or tasks. In addition, challenging tasks have been found to encourage learners to utilize their metacognitive strategies (Babakhani, 2014; Ergulec & Agmaz, 2023). This is mainly manifested in students’ behavioral engagement in challenging academic activities, such as in the application and innovation of knowledge and activities that exceed the basic learning requirements. Another crucial factor for successful online learning is the question of whether appropriate learning strategies are adopted (Zhu & Doo, 2022). Self-monitoring is closely related to learners’ cognitive and metacognitive processes. According to Garrison (1997), self-monitoring refers to learners’ responsibility for the construction of their personal learning, encompassing cognitive and metacognitive processes. The engagement of self-monitoring and management in online learning can also reflect the extent of students’ engagement in clarifying learning tasks and requirements. Therefore, “interaction”, “challenge”, and “self-monitoring” were recognized as three key behaviors affecting online learning engagement and were defined as evaluation indicators.

Through a comprehensive review and in-depth analysis of relevant studies in the field of learning engagement, following a meticulous process of extraction, classification, and integration, the key factors influencing online learning behavioral engagement were determined and the preliminary constituent indicators for online learning behavioral engagement were established. Specifically, there are five key factors: participation, focus, interaction, challenge, and self-monitoring. The factor of “participation” was decomposed into three indicators: log-in rate, input time, and access to learning resources. “Focus” is further specified by two indicators: the degree of single-task completion and long-term learning. “Interaction” is divided into three indicators: active interaction, peer interaction, and teacher–student interaction. Similarly, “challenge” consists of task workload, the use of cognitive tools, and resource expansion. Finally, “self-monitoring” is divided into three indicators: clear learning objectives, self-evaluation and reflection, and the use of management tools.

#### 4.1.2. Revising the Evaluation Indicators

**Figure 2:**
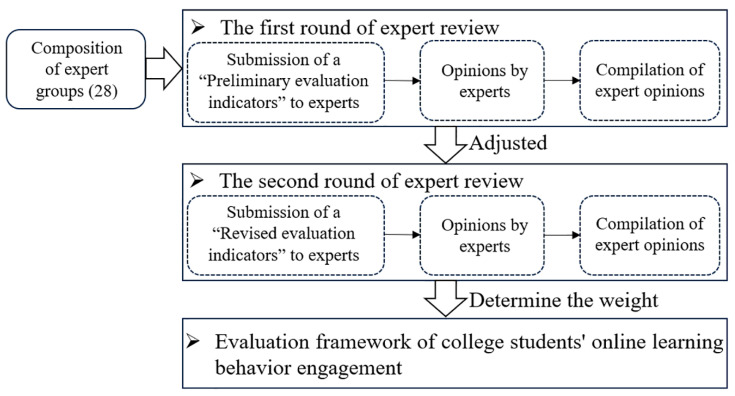
The process of applying the Delphi method to revise the evaluation framework.

Based on the application rules of the Delphi method, two rounds of expert consultations were conducted, leading to the development of an evaluation framework for college students’ engagement in online learning behaviors. Specifically, the process of applying the Delphi method to revise the evaluation framework is shown in Figure 2.

1. Expert composition and questionnaire design

In selecting consulting experts, the panel was composed of university professors with online teaching experience and professional researchers in the field of online learning engagement. Based on this criterion, a total of 28 experts were invited to participate in the process of refining the evaluation framework. Their responsibilities included evaluating the indicators at each level, assessing their significance within the indicators, and making necessary revisions and improvements.

2. Processes of expert consultation and evaluation

(1) The first round of expert review

During the initial round of expert consultation, 28 experts were invited, and 26 valid questionnaires were obtained. Based on the evaluations provided by the experts, the mean value, standard deviation, and coefficient of variation for each indicator were computed. Subsequently, modifications were carried out in accordance with the expert feedback. Specifically, indicators with similar or redundant meanings were either deleted or merged, the missing indicators suggested by the experts were added, and the indicators with unclear expressions were redefined. The key modifications are summarized as follows.

The mean values of the five indicators ranged from 4.00 to 4.57, while the standard deviations were between 0.05 and 0.27, and the coefficients of variation were between 0.01 and 0.06. These five indicators were of high importance, as evidenced by the concentrated expert opinions, high level of coordination, and effective convergence. Hence, all five indicators were retained.

A consensus was reached among experts to modify the secondary indicator “clear learning objectives” to “make learning plans”, thus necessitating the relevant adjustment.

Under the “focus” indicator, in line with the experts’ recommendations, the second-level indicator “task completion per unit time” was incorporated.

The secondary indicators “active interaction” and “peer interaction” were considered to have overlapping meanings, resulting in suggestions for modifying their expressions.

Experts emphasized that the “learning plan completion degree” is a critical indicator reflecting “self-monitoring”, leading to the addition of this secondary indicator under the primary indicator of “self-monitoring”.

Expert feedback pointed out an issue with the expression “expanding resources”, which was then revised to “expand the use of resources”, and consequently, the indicator content was adjusted. In summary, after the initial expert review, the evaluation indicators consist of 5 primary indicators and 16 secondary indicators.

(2) The second round of expert review

Based on the findings from the first round, modifications were implemented in the evaluation framework, which led to the development of the second round of the expert consultation questionnaire. This questionnaire incorporated the average scores, standard deviations, and coefficients of variation obtained from the initial expert consultation, as well as the expert judgments. An examination of the retrieved expert evaluation data led to the following specific revisions.

The average values of the 16 secondary indicators ranged from 3.70 to 4.80. The standard deviations fell between 0.40 and 0.87, and the coefficients of variation were between 0.08 and 0.23, suggesting the high significance of these evaluation indicators.

Standard deviations, all being less than 1, demonstrated that after the initial round of expert consultation, the opinions tended to converge.

3. Determining the weights of evaluation indicators

In this context, weight represents the proportional importance of each factor within the comprehensive evaluation framework. This study employed the Delphi method and AHP for the determination of the weights of the evaluation indicators.

(1) Building a hierarchical model

The evaluation framework was organized into three tiers: the target layer (A), the criterion layer (B), and the alternative scheme layer (C). This hierarchical structure constituted the model for assessing the online learning behavior engagement of college students.

(2) Constructing judgment matrix

In accordance with the hierarchical model, two judgment matrices were established. The first matrix pertained to pairwise comparisons conducted between each indicator within the criterion layer B and the target layer A. The second matrix involved pairwise comparisons of the alternative scheme layer C with respect to the same level indicators in the criterion layer B.

(3) Consistency test and weight calculation

Expert scoring data were inputted into the AHP software (version number: 0.5.2) for group decision-making calculations. This process included determining the weight of each indicator, computing the maximum eigenvector (wi) for each judgment matrix, identifying the maximum characteristic root (λ Max), and evaluating the consistency ratio (Cr). For example, take the judgment matrix for the overall goal of “behavioral engagement A”, which incorporated the primary indicators: “participation B1”, “focus B2”, “interaction B3”, “challenge B4”, and “self-monitoring B5”. The consistency ratio of this matrix was Cr = 0.0412 < 0.1, demonstrating that the judgment matrix successfully passed the consistency test. The final results of the indicators’ weights are shown in Table 1.

### 4.2. The Formation of Online Learning Behavioral Engagement Evaluation Framework for College Students

After two rounds of incorporating expert opinions, the definitive evaluation framework for college students’ online learning engagement was formulated. This framework functioned under a general objective and consisted of 5 primary indicators and 16 secondary indicators. Stringent consistency tests were conducted on all matrices to guarantee the dependability of the evaluation indicators. The comprehensive weights for each constituent are displayed in Table 2.

## 5. Data Collection and Procedures

### 5.1. Data Collection

In accordance with the requirements of empirical research, third-year undergraduate students majoring in Educational Technology at Northwest Normal University (located in Lanzhou City, Gansu Province in western China) were selected as the participants for the experiment. Before the commencement of the research, informed consent was obtained from the research subjects. After signing the informed consent forms, they participated in the research process on a voluntary basis. A total of 63 students from two experimental classes were involved. Among them, there were 27 students in Class 1 (labeled S1 to S27) and 36 students in Class 2 (labeled S28 to S63). All participants had prior experience in online learning and boasted a relatively good level of digital literacy. During the process of participating in the research, the subjects were marked throughout to ensure their anonymity.

The online teaching period lasted from 28 August 2019 to 28 November 2019, spanning three months. During this period, online learning behavior data for all students in both classes were collected. The online course involved in the experiment was “Mobile Application Design and Development (Frontend)”, which balanced theoretical learning with practical application. The instructor primarily conducted interactive teaching in traditional classroom settings, using an online learning platform for “online” interactions with students. Outside of class, students engaged in autonomous online learning through the platform. The “Ketangpai” online learning platform was selected as the designated experimental environment. This platform supported various functions, including online testing, posting topics or announcements, tracking attendance, and storing online teaching resources, as well as supporting features such as online classroom interaction, grade management, and teaching data visualization. It systematically collected students’ online learning behavior data. However, due to certain functional limitations, some online behavior data could not be fully collected. For instance, the platform was temporarily unable to collect interaction behavior data including “active interaction”, “peer interaction”, and “teacher-student interaction”.

### 5.2. Data Processing Methods

This study analyzed and conducted research based on all the online behavior data that could be collected within the experimental environment. The online learning behavior data from the selected experimental classes were relatively complete, and the online learning activities were highly representative. Specifically, the sources of data and the corresponding data processing methods are presented in Table 3.

## 6. Data Analysis

In this study, various online learning behaviors are considered as independent variables, while the total coursework grades (academic performance) are regarded as dependent variables for exploring the relationships between multiple independent and dependent variables. Based on the data sources of college students’ online learning behaviors collected from the online course platform “Classroom Pie”, descriptive statistical analyses, correlation analyses, and predictive analyses were performed. Firstly, students from Class 1 and Class 2 were taken as samples for analysis. By computing the ‘login rate’ and ‘interactive test participation rate’ of the two classes, the overall characteristics of college students’ online learning behaviors were analyzed, and the differences in online learning between the two classes were explored. Subsequently, all the data sources of the two classes were combined to analyze the correlation between the assessment indicators and academic performance, aiming to investigate the correlation between online learning behavior indicators and academic performance. Finally, the three online behavior indicators with the highest correlation with academic performance were selected, and regression prediction analyses were carried out with academic performance to explore the regression equation model of effective online learning behavior indicators for predicting online academic performance.

## 7. Results

### 7.1. Overall Characterization of Online Learning Behaviors

#### 7.1.1. Log-In Learning Behavior Analysis

**Figure 3:**
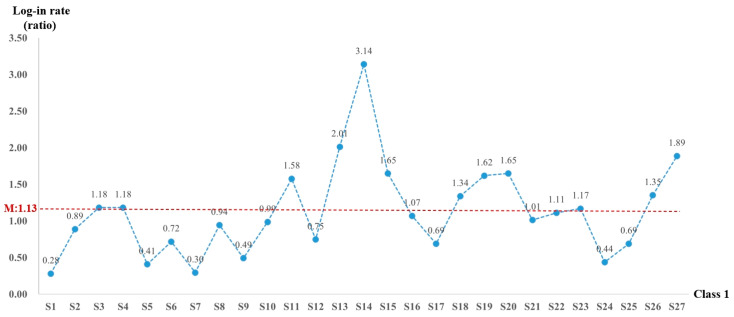
Log-in rate of students in Class 1.

**Figure 4:**
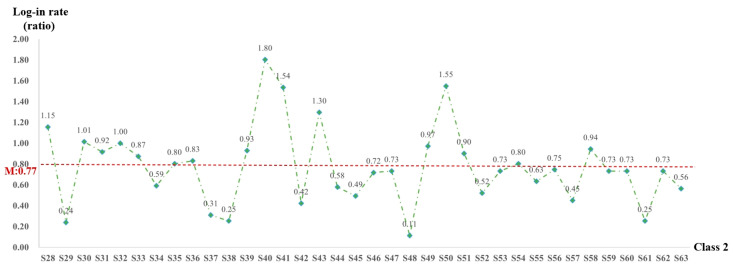
Log-in rate of students in Class 2.

The behavior of students logging into the online learning platform is a crucial indicator of their engagement and motivational commitment in the learning process. During the study, online courses lasting for 71 days were monitored, with weekends excluded during the initial phase (from Monday to Friday). The total number of log-ins on the course platform was counted, and the ratio of log-ins to study days was calculated to obtain the online learning log-in rate. This rate serves as a key metric for analyzing students’ engagement in online learning. The log-in rates for both classes are shown in Figure 3 and Figure 4.

As depicted in Figure 3, the average log-in rate for Class 1 is 1.13. It is worthy of note that 12 students (S3, S4, S11, S13, S14, S15, S18, S19, S20, S23, S26, and S27) displayed a log-in rate higher than the average of the class whereas the remaining 15 students had a rate lower than the average. Student S14 was particularly prominent, with a significantly high rate of 3.14, in contrast to S1, who had the lowest rate of 0.28.

As shown in Figure 4, the average log-in rate for Class 2 was 0.77. It is notable that 16 students (S28, S30, S31, S32, S33, S35, S36, S39, S40, S41, S43, S49, S50, S51, S54, and S58) exceeded the average of the class. Among them, student S40 had a significantly higher rate of 1.80 compared to the other classmates. Conversely, the remaining 20 students had a log-in rate lower than the class average.

When comparing the log-in rates of the two classes, it is evident that Class 1 had a significantly higher average log-in rate compared to Class 2. Furthermore, the students with the highest log-in rates in Class 1 considerably exceeded those in Class 2. In Class 1, a greater proportion of students exceeded the average log-in rate of their class in contrast to Class 2. Specifically, 55.5% of the students in Class 1 (15 out of 27) displayed a log-in rate higher than 1.0, indicating that they logged into the platform at least once a day. On the other hand, only 19.4% of the students in Class 2 (7 out of 36) had a log-in rate higher than 1.0. This disparity highlights that the overall online log-in rate and the level of commitment to online learning among students in Class 1 were substantially higher than those in Class 2. This difference was directly related to the fact that Class 2 consisted of a large number of ethnic minority students who had a relatively lower level of learning initiative.

#### 7.1.2. Participation Rate Analysis of Interactive Test Questions

**Figure 5:**
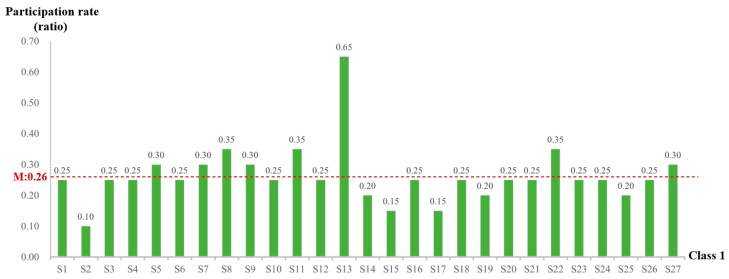
Participation rate of Class 1 students in the “interactive test”.

**Figure 6:**
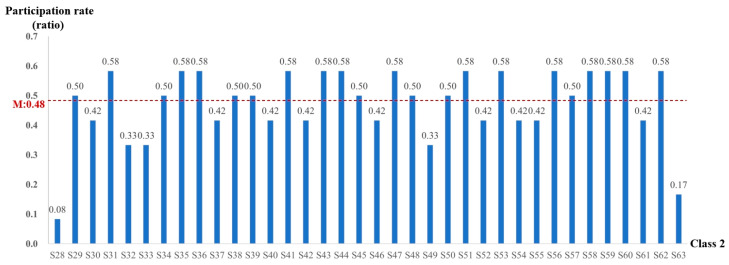
Participation rate of students in Class 2 in “interactive test”.

Interaction holds a crucial position in the educational process as it promotes learners’ engagement with learning resources and assists in knowledge acquisition. In order to evaluate “learning program completion”, an analysis was carried out on students’ participation rates in online interactive test questions. In both classes combined, the instructor organized a total of 20 test interactions in Class 1 and 12 in Class 2. The participation rate, which was computed as the ratio of students’ involvement in test interactions to the total number of test interactions, was utilized for analysis. The participation rates for students in Class 1 and Class 2 are presented in Figure 5 and Figure 6, respectively.

Figure 5 shows that the average participation rate of Class 1 students in the “interactive test” was 0.26. Among them, eight students (S5, S7, S8, S9, S11, S13, S22, S27) had a participation rate higher than the class average while the remaining nineteen students had a rate lower than the average. Student S13 had a remarkable participation rate of 0.65, which significantly exceeded that of other students in the class. In addition, students S8, S11, and S22, each with a rate of 0.35, also showed relatively high participation rates. In contrast, student S2 had the lowest participation rate of 0.10.

As shown in Figure 6, the average participation rate of students in Class 2 in the “interactive test” was 0.48. It is notable that 22 students exceeded the class average. Among them, 14 students (S31, S35, S36, S41, S43, S44, S47, S51, S53, S56, S58) achieved a remarkable participation rate of 0.58, which significantly exceeded their class average. However, it is incorrect that student S28 had the highest participation rate in his class at 0.08 as the text previously stated that 14 students had a rate of 0.58. Students S59, S60, and S62 also achieved a participation rate of 0.58, standing out with significantly higher rates compared to their class average. Student S28 recorded the lowest participation rate in his class at 0.08.

A comprehensive comparative analysis of the two classes indicates that, on average, students in Class 2 demonstrated significantly higher participation rates in test questions in comparison to those in Class 1. Nevertheless, in terms of students with the highest participation rates in test questions, Class 1 outperformed Class 2. In Class 1, eight students, accounting for 29.6% of the class, exceeded the average participation rate, whereas in Class 2, the number increased to twenty-two students, representing 61.1% of the class. Overall, the participation rate of students in Class 2 was notably higher than that of Class 1.

Overall, although the students in Class 2 had a lower log-in rate, they strived to obtain higher course grades and corresponding credits by actively participating in online interactive test questions during their platform log-in sessions. Conversely, the students in Class 1 showed stronger initiative in online learning. They focused on interacting with the platform’s resources, mastering knowledge and skills, and completing the online interactive test questions provided by the instructors.

### 7.2. Correlation Analysis of Assessment Indicators and Academic Performance

To guarantee the relevance and educational significance of engagement indicators, this study concentrated on behavioral aspects relevant to academic performance. Subsequently, a correlation analysis was carried out using SPSS 25.0 software to explore the connection between these behavioral engagement measures and the students’ final academic results. By regarding academic performance as the dependent variable and online learning behavior indicators as independent variables, the study intended to determine the correlation coefficients and coefficients of determination. This method was used to identify significant relationships and evaluate the significance between variables. The result of this analysis is shown in Table 4, presenting the correlation between online behavioral indicators and academic performance.

Referring to Table 4, it is clear that with the exception of three indicators, namely, “Self-evaluation and reflection”, “Use of online management tools”, and “Expand resource usage online”, which showed no significant correlation with academic performance (*p* > 0.05), the remaining six indicators displayed varying degrees of significant correlation with academic outcomes. Specifically, “Visits of online learning resources” and “Long-term online learning” had strong correlations of 0.574 and 0.771, respectively, indicating a strong connection. These indicators, which represented the frequency of accessing online resources and continuous online learning beyond the average semester length, were strongly correlated with academic performance. The other three indicators, namely, “Completion rate of online test questions”, “Online engagement duration”, “Submission rate of online homework”, and “Log-in rate”, showed moderate correlations ranging from r_s_ = 0.315 to r_s_ = 0.432, with the order of magnitude being “Completion rate of online test questions” > “Online engagement duration” > “Submission rate of online homework” > “Log-in rate”.

The coefficient of determination (R^2^), obtained by squaring the correlation coefficient (r), clarifies the percentage of variation accounted for by a linear relationship. Indicators like “Long-term online learning” and “Visits of online learning resources”, which had strong correlations with course grades, explained 59.44% and 32.95% of the variance in academic performance, respectively. On the other hand, the four indicators with moderate correlations to academic performance accounted for between 9.92% and 18.66% of the variance in academic outcomes. Consequently, these results emphasized the significant role that certain online behavioral indicators play in predicting and explaining students’ academic achievements.

### 7.3. Predictive Analysis of Assessment Indicators and Academic Performance

By applying the Pearson coefficient correlation analysis method, we systematically eliminated indicator items that were not accessible on the online platform, as well as online learning behavior variables that had weak correlations with academic performance. Through this process, three online learning behavior variables that demonstrated a strong correlation with academic performance were identified: “Visits of online learning resources”, “Long-term online learning”, and “Completion rate of online test questions”.

**Figure 7:**
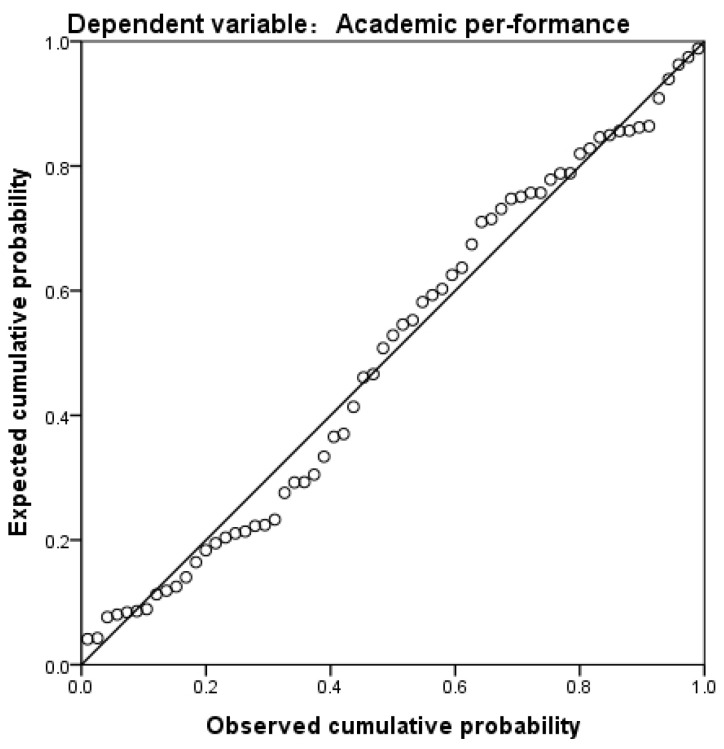
Normal P-P plot of the standardized residuals of the regression.

In light of the observed correlations, it was essential to determine the most influential behavioral variables that affected students’ academic performance from among several online learning measures. To achieve this, we carried out regression analyses using multiple linear regression methods in SPSS 25.0. This analysis aimed to uncover the most impactful online learning behavior variables and their corresponding predictive models for academic performance. Before performing the regression analysis, the variance and normality of the data were analyzed using SPSS 25.0 to assess the residual normality. The results indicated that the points on the P-P plot closely adhered to both sides of the diagonal, suggesting that the regression residuals approximated a normal distribution. Consequently, the research data met the assumptions of independence, homogeneity of variance, and normality. Specifically, the normal P-P plot of the standardized regression residuals is shown in Figure 7.

During the multiple linear regression analysis, “academic performance” was used as the dependent variable while the three previously identified effective online assessment behavior indicators served as independent variables. The results of the regression analysis parameter tests are presented in Table 5. This table contains the key findings derived from the regression analyses, providing insights into the effectiveness and predictive power of the selected online learning behavior indicators in relation to students’ academic performance.

The goodness of fit, represented by R, measures a model’s efficacy in conforming to the observed data. A value approaching 1 implies a better model fit. The adjusted R square, a more refined metric, provides a more precise evaluation. In our study, the R value was 0.822, demonstrating the good functionality of the regression equation. The adjusted R square, at 0.658, further validated that the regression model was well fitted, indicating that the independent variable explained 65.8% of the variance in the dependent variable. The *p*-value, recorded as 0.000 < 0.001, emphasized the significance of this regression equation’s predictive model.

Subsequently, by utilizing online learning behavior indicators that were strongly correlated with academic performance, we conducted multiple linear regression analysis with analytical software to develop a prediction model. The results of this regression model for academic performance are presented in Table 6. This table serves as a comprehensive resource, encapsulating the complex relationships between online learning behavior and its corresponding impact on academic performance.

The analysis results, as shown in Table 6, disclose insightful details regarding the impact of specific online learning behaviors on academic performance. The *p*-value related to the slope rate of online test questions was 0.089, exceeding the traditional threshold of 0.05. This implies that the independent variable, online test questions, did not have a statistically significant positive impact on the dependent variable, academic performance.

On the contrary, the *p*-values for the slope rates of “Visits of online learning resources” and “Long-term online learning” were 0.003 and 0.000, respectively, both less than 0.01. This indicates a highly significant linear relationship between these online learning behavior indicators and academic performance. The regression equation obtained from the analysis is expressed as Y (academic performance) = 64.543 + (Visits of online learning resources) × 0.03 + (Long-term learning) * 0.513. Although the robustness of this predictive model is remarkable, it requires refinement through an enlarged data set to enhance its application value further.

## 8. Discussion

### 8.1. Framework for Evaluating Online Learning Behavior Engagement: Exploring Influencing Factors from the Perspective of Weights

During the development process of the evaluation framework, two rounds of revisions were made to the initially drafted indicator system by utilizing the Delphi expert consultation method. Subsequently, weights were assigned to the determined indicators through the AHP. Eventually, the complete “Evaluation Framework for College Students’ Online Learning Behavioral Engagement” was developed. Notably, the weights allocated to the primary indices, namely “focus” and “self-monitoring”, were both greater than 0.3 independently. This suggests that when engaged in diverse online learning tasks, college students are capable of concentrating their attention on the learning content or tasks and conducting timely self-evaluation and reflection on certain issues arising during the online learning process. These characteristics have a positive impact on promoting college students’ engagement in online learning. As some researchers have noted, some professors may require students to turn on their cameras, which can enhance accountability and provide an incentive for students to visibly focus as they would in a traditional in-person classroom (Hollister et al., 2022).

An examination of the weights of the corresponding secondary indicators shows that the values for “Long-term online learning”, “Completion rate of online test questions”, and “Self-evaluation and reflection” exceeded 0.1. This highlights the critical importance of intrinsic motivation for students to engage in continuous online learning each time they access an online learning platform. As Samat et al. (2020) discovered in their study, intrinsic motivation is a predictor of a student’s intention to use online learning. Furthermore, increasing the completion rates of online test questions and reflecting on and evaluating task completion within the online learning environment are essential for enhancing engagement in online learning. Significantly, previous studies consistently demonstrated the positive impact of behavioral engagement on academic performance (Bråten et al., 2018). For instance, Morris et al. (2005) investigated student participation, duration, and frequency in online courses and identified a positive correlation between participation and academic performance.

Within the extant body of research, indicators related to homework, study duration, self-reflection, emotions, and metacognition are frequently analyzed as submodules within the comprehensive framework of learning engagement. Numerous studies have emphasized the importance of these factors in guaranteeing effective learning outcomes (Vo & Ho, 2024; Zheng et al., 2023). The weights of the evaluation indicators disclosed in this study provide valuable perspectives for probing into college students’ online learning engagement from a psychological standpoint. This might entail the design of online learning activities that are customized according to the psychological traits of college students. Additionally, it could involve exploring strategies for improving self-awareness, reflection, and regulation during online learning, as well as examining the influence of these behavioral indicators on emotional engagement. These areas offer fruitful directions for future research initiatives.

### 8.2. Enhancing Engagement in Online Learning: Analysis of Key Behavioral Indicators

The correlation analysis conducted for the evaluation indicators and academic performance yielded significant insights. It is notable that all six valid online assessment indicators displayed correlation coefficients greater than 0.3, indicating a substantial positive correlation at the intermediate or higher level with academic performance. Specifically, “Visits of online learning resources”, and “Long-term online learning” presented strong correlation coefficients, both exceeding 0.6. This implies that students can markedly enhance their academic achievement by engaging in long-term online learning while utilizing course resources. Indeed, studies have found that students exert greater effort, enthusiasm, and commitment to online learning when they are presented with relevant tasks and learning materials (Alamri et al., 2020; Chiu, 2022; Martin et al., 2018). Simultaneously, the other four indicators, namely “Completion rate of online test questions”, “Online engagement duration”, “Submission rate of online homework”, and “Log-in rate”, showed moderate correlations with academic performance.

These findings are in accordance with the research carried out by other scholars. Specifically, the correlation between extended participation in learning activities and enhanced academic performance has been thoroughly documented in the literature (Bravo-Agapito et al., 2021; Yokoyama, 2019). Empirical studies have provided evidence for the positive influence of accessing online resources or platforms on students’ academic performance. Furthermore, a delay in accessing online learning materials has been associated with lower final grades (Alghamdi, 2013; Suppan et al., 2021). Previous research consistently emphasized the significance of student engagement for academic achievement, indicating that engaged students were more likely to exhibit perseverance, self-regulate their behavior to attain goals, and obtain enjoyment from learning and challenges (Klem & Connell, 2004).

This analysis highlights the significance of investigating the relationship between online learning behavioral engagement and elements such as self-cognition, self-monitoring, and regulation. Stimulating the intrinsic motivation and perseverance of college students in online learning, facilitating a deeper interaction with online learning resources, and effectively organizing online learning time are identified as crucial factors for improving academic performance among college students. The identified correlations suggest potential intervention approaches for enhancing students’ engagement with online learning platforms, thereby positively influencing their academic outcomes.

The coefficient of determination (R^2^) serves as an indicator of the proportion that can be explained within the linear relationship or the line of best fit. The results of this study clearly demonstrate that two key indicators, namely “Visits of online learning resources” and “Long-term online learning”, which have significant associations with academic performance, jointly account for over 30% of the variance in academic outcomes. In contrast, the remaining four indicators, which have a moderate relationship with academic performance, together explain 10% to 19% of the variance in academic results. This emphasizes the profound impact of the time that college students spend on online learning during each platform log-in while using digital learning resources on their overall online academic performance.

To effectively enhance college students’ engagement in online learning, instructors should carefully select high-quality learning resources within their platforms, ensuring they are in line with students’ cognitive abilities. Moreover, ensuring an appropriate duration for students’ online learning during each log-in is essential for promoting sustained engagement in online learning. Extensive research emphasizes the importance of tailoring online learning to meet the specific needs of students (Liu et al., 2023). Transferring an existing in-person course to an online learning platform requires careful consideration, necessitating the alignment of course content and design with student requirements (Aristovnik et al., 2020; Baltà-Salvador et al., 2021). Therefore, this paper presents new perspectives on the development of digital resources for promoting college students’ engagement in online learning behaviors. Additionally, it proposes intervention strategies to ensure the provision of high-quality online learning hours. These insights make a significant contribution to the ongoing discussion on optimizing online learning experiences, highlighting the need for customized resources and instructional designs that match student preferences and requirements.

## 9. Limitations and Future Directions

Although this study offered valuable insights, it is essential to recognize its limitations and suggest directions for future research. One of the limitations lay in the relatively small and homogenous sample size, which may have limited the generalizability of the research findings. To enhance the generalizability and robustness of the proposed online learning behavior engagement evaluation framework and the constructed academic performance prediction model, future research should give priority to increasing the sample size. By doing so, a more comprehensive understanding of college students’ online learning behavior engagement levels can be achieved. Meanwhile, this study was carried out before the outbreak of the pandemic, and the corresponding research data were collected simultaneously. The research conclusions are mainly applicable to college students’ regular online learning. If this study had been conducted during the pandemic, with the increase in the frequency of online interactive activities and the enrichment of online resources, the collected online learning behavior data would have been more diversified, which would have been more conducive to exploring the influencing factors of college students’ online learning engagement. This also expands the main research tasks for the future.

Second, since college students’ engagement in online learning is affected by multiple factors, future research efforts should adopt a multifaceted approach that combines multi-factor analysis with qualitative research designs. This strategy will facilitate a detailed exploration of the various factors influencing college students’ engagement in online learning behaviors and inform the development of targeted intervention strategies. Furthermore, due to the limited data collection capabilities of the experimental platform, it was challenging to collect all types of interaction behavior data from when students participate on an online learning platform. With the continuous development of digital technology and the continuous maturation of online learning platforms, the student online learning behavior data that can be collected will become richer, providing support for a more in-depth exploration of college students’ online learning engagement. As a result, this research has not yet explored the relationship between online interaction and learning engagement, which will be the main research focus in future work to effectively enhance students’ online learning engagement.

Finally, to improve the accuracy of the prediction model, it is also necessary to enhance the diversity of teaching activities and enrich the multi-source collection of online learning behavior data during the online teaching process. This includes promoting the recording and monitoring of various types of data throughout students’ participation in online learning and forming a three-dimensional learner-centered data set from multidimensional information such as online learning activities, social networks, self-knowledge and monitoring, self-reflection, and regulation. By developing an assessment model of online learning behavior, this paper aims to provide a theoretical framework for a more comprehensive assessment of college students’ commitment to online learning and the prediction of online learning effectiveness. Subsequent research efforts should further explore the intricate relationships between college students’ cognitive engagement, emotional engagement, motivation, self-efficacy, and other aspects of online learning engagement.

Moreover, future research could adopt a psychological perspective to explore the intrinsic relationships between online learning engagement and self-cognition, self-control, self-reflection, and self-regulation. This line of inquiry will contribute to a deeper understanding of the psychological mechanisms underlying online learning engagement. As Reyes-de-Cózar et al. (2023) pointed out, emotional engagement is linked to students’ positive responses related to educational institutions, their learning, and interactions between teachers and peer; cognitive engagement relates to scenarios in which students can develop strategies for the self-management of their learning. At the same time, it is essential to examine strategies and mechanisms for enhancing college students’ online learning engagement from the perspective of its constituent dimensions. For example, in terms of cognitive engagement, research could investigate the effects, interventions, and underlying mechanisms involving various cognitive strategies and cognitive engagement; in terms of emotional engagement, studies could explore the impact mechanisms involving different emotional experiences and emotional engagement. Additionally, research could broaden its focus to include intervention strategies and activity designs aimed at optimizing the online learning experience to effectively improve the quality of online learning for college students. This multidimensional approach will contribute to a more comprehensive understanding of online learning engagement and support ongoing efforts to improve the overall quality of online education for college students.

## 10. Conclusions

Drawing upon an extensive literature review, five crucial factors influencing college students’ engagement in online learning behaviors along with 16 corresponding data collection indicators were collated and summarized. This process encompassed careful and simplified screening, consultation with experts, and confirmation through the AHP. Subsequently, the “Evaluation framework of college students’ online learning behavior engagement” was established. By employing this comprehensive evaluation framework, experimental classes were meticulously chosen, behavior data related to learning engagement were collected, and a detailed assessment of online learning behavioral engagement was conducted. Through this empirical study, the correlation between online learning behavior indicators and academic performance was explored and the predictive capabilities of effective behavioral engagement indicators on academic outcomes were further scrutinized.

As the data collection methodology and the items collected on the online learning platform continue to evolve, online learning behavior data is anticipated to become more abundant. This will facilitate a more comprehensive and accurate evaluation of students’ online learning behavioral engagement in accordance with the established model. The continuous improvement of data collection methods guarantees that future evaluations will be founded on a more profound understanding of the complex relationship between online learning behaviors and academic performance. Looking forward, the continuous enhancement of data collection practices will contribute to refining and deepening our comprehension of how students interact with online learning platforms. This will provide a foundation for future research and interventions aimed at optimizing the online learning experiences of college students.

## Figures and Tables

**Table 1:** Calculation results of B1–B5 judgment matrix.

A (Behavioral Engagement)	B1 (Participation)	B2 (Focus)	B3 (Interaction)	B4 (Challenge)	B5 (Self-Monitoring)	Weight
B1 (Participation)	1	0.3567	0.7920	1.9841	0.3637	0.1202
B2 (Focus)	2.8034	1	2.2204	5.5623	1.0195	0.3370
B3 (Interaction)	1.2626	0.4504	1	2.5051	0.4591	0.1518
B4 (Challenge)	0.5040	0.1798	0.3992	1	0.1833	0.0606
B5 (Self-monitoring)	2.7498	0.9809	2.1780	5.4561	1	0.3305

**Table 2:** Evaluation framework of college students’ online learning behavior engagement.

Key Factors	Weight	Indicators	Weight	Indicators Description
Participation	0.1202	Log-in rate	0.0112	Frequency of log-in to online learning platform.
		Online engagement duration	0.0604	Average length of online learning platform log-in.
		Visits of online learning resources	0.0486	Frequency of browsing online learning resources.
Focus	0.3370	Submission rate of online homework	0.0532	Submission status of online homework.
		Long-term online learning	0.1326	Each time students logged in to the platform to participate in online learning was longer than the average number of times of their own learning time in the whole semester.
		Completion rate of online test questions	0.1512	The completion rate of online release test questions.
Interaction	0.1518	Active interaction	0.0735	Students’ own active participation in platform interactions.
		Peer interaction	0.0444	Students’ own participation in online peer interactions.
		Teacher–student interaction	0.0338	Online interaction between students and lecturers.
Challenge	0.0606	Completion rate of online challenge tasks	0.0260	Students’ completion of tasks beyond online learning requirements. For example, the total number of “rush to answer”.
		Use of online cognitive tools	0.0246	Students’ use of online information tools in order to access, save and process online information.
		Expanding resource usage online	0.0100	Overall download or browsing of online learning resources.
Self-monitoring	0.3305	Clear learning objectives	0.0807	Use of the learning plan creation module in the learning platform.
		Self-evaluation and reflection	0.1212	Self-judgement and evaluation of the completion of online learning tasks and problems and difficulties encountered.
		Use of online management tools	0.0384	Students’ use of online management tools that enhance learning efficiency.
		Completion of learning plan	0.0903	Overall completion of various tasks and programs in online learning.

**Table 3:** Various data sources and corresponding data processing methods.

Data Sources	Data Processing Methods
Log-in rate	Counted the number of visits to the platform.
Online engagement duration	Total time spent logging on the platform divided by the number of visits to the class.
Visits of online learning resources	The number of student interactions divided by the total number of Teacher Organizational interactions.
Submission rate of online homework	The number of student assignments submitted divided by the total number of assignments required to be submitted.
Long-term online learning	Each time students logged into the platform to participate in online learning is longer than the average number of times of their own learning time in the whole semester.
Completion rate of online test questions	Counted the number of completed test questions divided by the total number of published test questions.
Expanding resource usage online	Counted the total number of times students downloaded learning resources.
Self-evaluation and reflection	Total number of comments on announcements in the online learning platform.
Use of online management tools	Counted the total number of times students managed “Personal Portfolio”.

**Table 4:** Results of correlation analysis of online learning behavioral indicators and academic performance.

Dependent Variable	Online Learning Behavioral Indicators	N	Correlation Coefficient (r_s_)	Coefficient of Determination (R^2^)	*p*-Value
Academic performance	Log-in rate	63	0.315 *	0.0992	0.012
	Online engagement duration	63	0.377 **	0.1421	0.002
	Visits of online- learning resources	63	0.574 ***	0.3295	0.000
	Submission rate of online homework	63	0.318 *	0.1011	0.011
	Long-term online learning	63	0.771 ***	0.5944	0.000
	Completion rate of online test questions	63	0.432 ***	0.1866	0.000
	Self-evaluation and reflection	63	0.168	0.0282	0.188
	Use of online management tools	63	0.123	0.0151	0.335
	Expanding resource usage online	63	0.162	0.0262	0.204

Note: * *p* < 0.05, ** *p* < 0.01, *** *p* < 0.001.

**Table 5:** Parameter tests for regression analysis.

R	R^2^	Adjusted R^2^	Errors in Standardized Estimates	Durbin-Watson	F	*p*-Value
0.822	0.675	0.658	4.0936	1.364	40.835	0.000

**Table 6:** Results of the regression model analysis of effective behavioral engagement indicators of academic performance.

	Unstandardized Coefficient		Standardized Coefficient	t	*p*-Value	Covariance Statistics	
	B	Standard Error	β			Tolerances	VIF
(Constant)	64.543	5.002		12.904	0.000		
Visits of online learning resources	0.030	0.010	0.260	3.088	0.003	0.776	1.289
Long-term online learning	0.513	0.073	0.603	6.984	0.000	0.738	1.355
Completion rate of online test questions	9.620	5.569	0.139	1.727	0.089	0.850	1.177

## Data Availability

The raw data supporting the conclusions of this article have been made available at: DOI:10.17632/cx4764nmk6.1; https://data.mendeley.com/datasets/cx4764nmk6/1 (accessed on 6 January 2024).
